# Toxicological Assessment of a Lignin Core Nanoparticle Doped with Silver as an Alternative to Conventional Silver Core Nanoparticles

**DOI:** 10.3390/antibiotics7020040

**Published:** 2018-05-04

**Authors:** Cassandra E. Nix, Bryan J. Harper, Cathryn G. Conner, Alexander P. Richter, Orlin D. Velev, Stacey L. Harper

**Affiliations:** 1 Department of Environmental & Molecular Toxicology, Oregon State University, Corvallis, OR 97331, USA; nixc@oregonstate.edu (C.E.N.); Bryan.Harper@oregonstate.edu (B.J.H.); 2 Department of Chemical and Biomolecular Engineering, North Carolina State University, Raleigh, NC 27606, USA; cgconner@ncsu.edu (C.G.C.); aprichte@ncsu.edu (A.P.R.); odvelev@ncsu.edu (O.D.V.); 3 Oregon Nanoscience and Microtechnologies Institute, Corvallis, OR 97330, USA; 4 School of Chemical, Biological and Environmental Engineering, Oregon State University, Corvallis, OR 97331, USA

**Keywords:** nanotechnology, environmentally-friendly, pesticide, antimicrobial, zebrafish

## Abstract

Elevated levels of silver in the environment are anticipated with an increase in silver nanoparticle (AgNP) production and use in consumer products. To potentially reduce the burden of silver ion release from conventional solid core AgNPs, a lignin-core particle doped with silver ions and surface-stabilized with a polycationic electrolyte layer was engineered. Our objective was to determine whether any of the formulation components elicit toxicological responses using embryonic zebrafish. Ionic silver and free surface stabilizer were the most toxic constituents, although when associated separately or together with the lignin core particles, the toxicity of the formulations decreased significantly. The overall toxicity of lignin formulations containing silver was similar to other studies on a silver mass basis, and led to a significantly higher prevalence of uninflated swim bladder and yolk sac edema. Comparative analysis of dialyzed samples which had leached their loosely bound Ag^+^, showed a significant increase in mortality immediately after dialysis, in addition to eliciting significant increases in types of sublethal responses relative to the freshly prepared non-dialyzed samples. ICP-OES/MS analysis indicated that silver ion release from the particle into solution was continuous, and the rate of release differed when the surface stabilizer was not present. Overall, our study indicates that the lignin core is an effective alternative to conventional solid core AgNPs for potentially reducing the burden of silver released into the environment from a variety of consumer products.

## 1. Introduction

Silver nanoparticles (AgNPs) are an effective antimicrobial agent and the most widely commercialized engineered nanomaterial, incorporated into half of all reported consumer and medical products in the Nanotechnology Consumer Products Inventory [1]. Prominent examples include cosmetics, clothing, shoes, detergents, water filters, phones, laptops, and toys [2,3,4]. AgNP use has risen steadily in the past decade (~52 new consumer products per year), and global production is estimated based on surveys of European producers to be between 12–1216 tons per year by 2020, assuming the number of products on the market continues to increase at the current rate [5,6]. With the increasing production and use of AgNPs, the fate and the subsequent release of silver in nanomaterial and ionic form into the environment are of concern. 

Research indicates that AgNPs can enter aqueous environments from discharges at the point of production, by erosion from household products, and from disposal of silver-containing products [7,8,9,10,11]. These studies have prompted the investigation of AgNP interactions in the environment [12], particularly aquatic systems, to determine which general intrinsic and extrinsic properties are important in determining fate [9,13,14,15]. Extrinsic properties include environmental factors and processes that can impact the fate of the particles in aquatic systems, such as pH, temperature, ionic strength of the water, and natural organic matter, as well as processes like sedimentation, deposition, dissolution, agglomeration, and/or particle sulfidation [16,17,18,19]. Intrinsic factors address inherent particle characteristics, such as size, shape, chemical composition, surface structure, and surface charge [12,20,21,22,23]. Extrinsic factors can interact with intrinsic features of nanoparticles to alter particle behavior with concomitant effects on properties, such as the bioavailability of AgNPs to living organisms; thus, a more comprehensive understanding is needed [13,24].

AgNPs are known to be toxic to many aquatic organisms, including algae, bacteria, invertebrates, and fish [2]. Several mechanisms of action have been proposed, mainly attributing the toxicity of AgNPs to silver ions released from the nanoparticle. However, nanoparticle-specific mechanisms are also being investigated, with data suggesting that mechanistic differences exist compared to dissolved silver [5,25]. Silver ion specific mechanisms include interactions with thiols and electron donor groups, which can impact enzymes and DNA, which makes them unavailable for cellular processes [26,27,28], denaturing of DNA and RNA, which ultimately affects protein synthesis [29,30], and production of superoxide radicals and other reactive oxygen species via reactions with oxygen [29]. Particle-specific mechanisms have been suggested that focus on the ability of AgNPs to cause cell membrane damage, leading to disruption in the ion efflux system in cells [31,32], as well as by intracellular ion release elicited by the acidic conditions of the lysosomal cellular compartment where particles are internalized (Trojan horse effect) [33]. Since multiple aquatic organisms may be at risk due to an increased prevalence of silver in the environment, it is important to consider ways to reduce the environmental silver burden related to AgNP production and use.

By applying the principles of green chemistry during nanomaterial design and synthesis, harmful effects to the environment can be limited while maintaining the desired antimicrobial activity [34]. In order to reduce silver ion release into the environment, a silver-doped lignin nanoparticle was engineered, which is anticipated to have lower environmental impacts upon release into the environment [35]. During the synthesis of these particles, we replaced the silver core with lignin, which was chosen as it is a natural biodegradable biopolymer [36]. Similar synthesized lignin nanoparticles have been shown to have no impact on algae and yeast survival, suggesting they have a high level of biocompatibility [37]. The lignin is easily precipitated into nanosized particles using environmentally-friendly solvents, and the resulting nanoparticles can be infused with up to ten times lower (Ag^+^) than silver core nanoparticles, and still maintain the same antimicrobial efficacy [35]. The particles are then surface-functionalized with a polycationic electrolyte layer to stabilize the particle, as well as to provide additional antimicrobial impact. The lignin nanoparticles exhibit both high and low affinity binding regions for silver ions, and these differing affinities, as well as the electrostatic barrier provided by the surface stabilizer, impact the rate of silver ion release to the surrounding solution [35,36]. It is expected that the low affinity binding sites will primarily release the majority of the weakly bound silver in the first 24 h [35]; however, we also wanted to investigate the long-term release from the high affinity binding sites, so two of the formulated samples were dialyzed to remove the weakly bound silver. When compared to their non-dialyzed counterparts, this allowed us to determine whether there are any differences in toxicological responses after the release of the loosely bound silver ions to quantify the potential environmental risks of these particles. 

Our aim was to elucidate which aspects of the formulation contribute most to the toxicity of the formulation, and to discover whether these nanoparticles exhibit any toxicity after dialysis, which intended to simulate post-consumer use. We hypothesized that (1) released silver ions from the lignin particle and the surface stabilizer are the main contributors to the aquatic toxicity of these nanoparticles; and (2) once the particles have been dialyzed to remove the ionic silver from the low affinity lignin-binding sites, there would be a reduction in toxicity of the formulated particles. To test these hypotheses, we utilized the embryonic zebrafish assay, which is a widely-used model for toxicity testing as it provides a suite of developmental endpoints that are critical to the survival of the organism [38,39]. Zebrafish also develop quickly and are optically transparent, which allows for easy observations of phenotypic responses [38]. Additionally, they share similar homology to humans, so observed effects of chemical stressors from this assay can potentially be extrapolated to human physiological responses [40].

## 2. Materials and Methods

### 2.1. Materials and Characterization

Reference component solutions of silver nitrate (AgNO_3_) salt (CAS# 7761-88-8, Fisher Scientific, Hampton, NH, USA) at 50 mg/L of Ag^+^ dissolved in ultrapure water and poly (diallyldimethylammonium chloride) (PDAC, MW 100,000–200,000, CAS# 26062-79-3, Sigma Aldrich, St. Louis, MO, USA) at 200 mg/L in ultrapure water were prepared and refrigerated at 4 °C until use. The lignin (Indulin AT) for the nanoparticle core was extracted from biomass as a by-product of kraft pulping processes [36,41]. The Indulin AT lignin powder (lot MB05) and supporting documentation were obtained from MeadWestVaco, SC. The size range of the particles after synthesis with the pH-drop flash precipitation method (Figure S1c) was 84 ± 5 nm in ultrapure water [35]. PDAC was chosen to provide a cationic surface charge to the particles, such that they would be attracted to the negatively charged bacterial cell membranes. Stock nanomaterial suspensions of the lignin nanoparticle (NP), the silver functionalized lignin nanoparticle (NP + Ag), the silver functionalized particle with the cationic PDAC surface (NP + Ag + PDAC), and the lignin nanoparticle with PDAC alone (NP + PDAC) were prepared as previously described, and tested for antimicrobial efficacy, as is reported in previous publications [35,36]. Stock concentrations of each component were as follows: 500 mg/L lignin nanoparticle, 5 mg/L Ag^+^, and 200 mg/L PDAC. All stock materials were stored in distilled water at 4 °C until use. Seven-fold dilutions of stock nanomaterial suspensions were performed with fishwater to prepare the varied exposure solutions. Fishwater was prepared by dissolving 260 mg/L Instant Ocean salts (Aquatic Ecosystems, Apopka, FL, USA) in reverse osmosis water and adjusting pH to 7.2 ± 0.2 using ~0.1 g sodium bicarbonate (conductivity 480–600 μS/cm) [39]. Experimental materials were stored under the same conditions as the reference materials. The NP + Ag and NP + PDAC formulations were solely used for comparative purposes, whereas the NP + Ag + PDAC is the proposed complete product formulation.

The samples to be dialyzed (NP + Ag and NP + Ag + PDAC) were placed in deionized water for 24 h which included a Slide-A-Lyzer MINI Dialysis Device (Thermo Scientific, Waltham, MA, USA) with a 10 K molecular weight cutoff membrane to remove dissolved silver from solution prior to dilution and testing. A second sample of NP + Ag was also dialyzed and allowed to age for 5 months prior to testing. Thus, the dialyzed samples included NP + Ag Aged, NP + Ag Fresh, and NP + Ag + PDAC Fresh, with the “Fresh” and “Aged” designations referring to when the sample was tested relative to when it was dialyzed.

The hydrodynamic diameter (HDD) and the zeta potential of each formulation that contained particles were measured in triplicate using a Zetasizer Nano ZS (Malvern Instruments Ltd., Worcestershire, UK) at 26.8 °C after dilution with fishwater to 50 mg/L. Aliquots (1 mL) were stored in an incubator under the same conditions as the embryonic zebrafish, until ready for analysis. Measurements were made over a five-day period, which also included an initial measurement (Day 0) which correlates with the exposure time of the experiment. Metadata associated with the zeta potential measurements can be found in Table S1.

### 2.2. Embryonic Zebrafish Assay

Exposure solutions of reference and nanomaterial suspensions were dispensed into 96-well plates, with each row having 12 wells of a given concentration of test material. Each well was filled with 200 μL of test solution and one of the eight rows on the plate was reserved for fishwater alone as a control. Adult zebrafish (*Danio rerio*) were maintained at the Sinnhuber Aquatic Research Laboratory (SARL) at Oregon State University, Corvallis, OR, USA. Embryos received from SARL were approximately 6–8 h post-fertilization (hpf) and were inspected under a dissecting microscope to ensure viability and developmental stage, then placed individually into wells of a 96-well plate. The chorionic membrane surrounding the zebrafish was preserved. Two replicate exposures were conducted over two weeks for each material, which allowed us to have a total sample size of 24 fish per concentration, per material. After plating, the exposure wells were covered with Parafilm to reduce evaporation, and embryos were incubated at 26.8 °C under a 14:10 light/dark photoperiod.

### 2.3. Toxicological Evaluations of Embryonic Zebrafish

Fish were observed at 24 hpf and 120 hpf for mortality, as well as a suite of developmental, morphological, and physiological abnormalities. At 24 hpf, embryos were evaluated for mortality, presence of spontaneous movement, delayed developmental progression, and notochord malformations. At 120 hpf, mortality was evaluated in conjunction with malformations of the snout, brain, pectoral and caudal fin, eye, jaw, otic structures, axis, trunk, somites, swim bladder, and body pigmentation. In addition, physiological and behavioral endpoints evaluated at 120 hpf include the presence of pericardial or yolk sac edema, impaired circulation and active touch response [39]. Hatching success was measured between 48 and 120 hpf, with embryos that hatched between 48 and 72 hpf being considered normal, and any individuals hatching after 72 hpf were considered delayed [42]. All endpoints were reported as either absent or present. Representative images of control fish and any individuals that displayed developmental abnormalities at 24 and 120 hpf were taken with an Olympus SZX10 microscope (Tokyo, Japan) fitted with an Olympus SC100 high resolution digital color camera (Olympus Corporation, Center Valley, PA, USA), and representative images are included in the Supplementary Materials (Figure S1). All experiments were performed in compliance with national care and use guidelines, and approved by the Institutional Animal Care and Use Committee (IACUC) at Oregon State University (ACUP #4764).

### 2.4. Measurement of Dissolved Silver and Particle-Associated Silver

Both the concentration of dissolved silver released from the nanoparticles and the silver associated with the particle itself were quantified by inductively coupled plasma-optical emission spectroscopy or mass spectrometry (ICP-OES or ICP-MS). To quantify silver content in solution, acid digestion of particles was performed using established methods [18,43]. Triplicate 0.5 mL samples of stock suspensions were centrifuged at 13,000× *g* for 10 min in a 3 kDa centrifugal filter (VWR, Radnor, PA, USA) with a polyethersulfone (PES) membrane, to separate the lignin particles from the filtrate. A total of 0.45 mL of filtrate sample was collected, diluted 10-fold with ultrapure water, and adjusted with 70% trace-metal grade HNO_3_ to a final concentration of 3% HNO_3_. For the lignin particle digestion, 0.1 mL of stock solution was digested in the same manner as the filtrate samples, without the centrifugation step. All samples were digested in Teflon tubes at 200 °C with 3 mL 70% trace-metal grade HNO_3_. The acid was allowed to completely evaporate, and the process was repeated three times. Final digested samples were dissolved in 5 mL of 3% HNO_3_ prior to ICP-OES/MS analysis. The silver ICP standard was purchased from RICCA Chemical Company (Ricca Chemical Company, Arlington, TX, USA) and diluted to six concentrations spanning the expected concentrations. All samples, including standards, were analyzed in triplicate with ICP-OES (Teledyne Leeman Labs, Hudson, NH, USA) for silver content, except the filtrate from the NP + Ag + PDAC sample, which was analyzed by ICP-MS (Thermo-Fisher, Waltham, MA, USA) to provide a lower level of detection (≥5 µg/L).

### 2.5. Statistical Analyses

All statistical analyses were conducted with SigmaPlot version 13.0 (Systat Software, San Jose, CA, USA), unless otherwise noted, and all differences were considered significant at *p* ≤ 0.05. For measurements of zeta potential and HDD, significant differences were determined with repeated measures analysis of variance (ANOVA) and Tukey’s post hoc analysis. Two-way ANOVA was conducted to ensure that there was no significant difference in mortality between replicate exposure plates prior to pooling of the data. Concentration–response curves were generated with the Environmental Protection Agency’s Toxicity Relationship Analysis Program (EPA TRAP v. 1.30, March 2015). EPA TRAP was also used to calculate the concentration at which fifty percent of exposed zebrafish perished (LC_50_) for each material, and the Litchfield/Wilcoxon formula was utilized for LC_50_ comparisons between treatments [44]. Significant sublethal endpoints were determined by Fisher’s Exact Test, by comparing the control (fishwater alone) response to each concentration response tested. To determine whether there were significant differences in the concentration of silver associated with the particle versus the filtrate in the ICP analysis, a paired *t*-test was utilized.

## 3. Results and Discussion

### 3.1. Particle Characterization

Average zeta potential and HDD for the formulated particles in fishwater were measured over a five-day period. As expected, the zeta potential of the formulated particles varied with the presence of the surface stabilizer with the lignin NP alone, and the NP + Ag, both having negative zeta potentials in fishwater (−24.6 and −29.0 mV, respectively), while the particles with the cationic surface stabilizer had positive zeta potentials with and without silver ions present (28.1 and 25.5 mV respectively. Over the five day incubation in fishwater, only relatively minor changes in particle zeta potential were identified (Figure S2a). Zeta potentials for the dialyzed particles in fishwater were similarly consistent over time, and correlated well with their non-dialyzed counterparts (Figure S2b).

The initial HDD of the various particle formulations in fishwater were similar to the 84 nm primary particle size ranging from 80 to 95 nm, depending on the particle type (Figure S2c). Most of the formulated particles had consistent HDDs over time, however, the NP + Ag sample had the largest increase in size, reaching 124 nm by the end of the five day incubation. The dialyzed NP + Ag samples (both freshly dialyzed and aged dialyzed samples) had much more consistent HDD than the non-dialyzed counterparts (Figure S2d). These data suggest that the rapid loss of silver ions from the non-dialyzed components to the solution can lead to some particle swelling, however, the surface stabilizer effectively stabilized the particles from agglomeration.

### 3.2. Analysis of Dissolved Silver and Particle-Associated Silver

**Figure 1:**
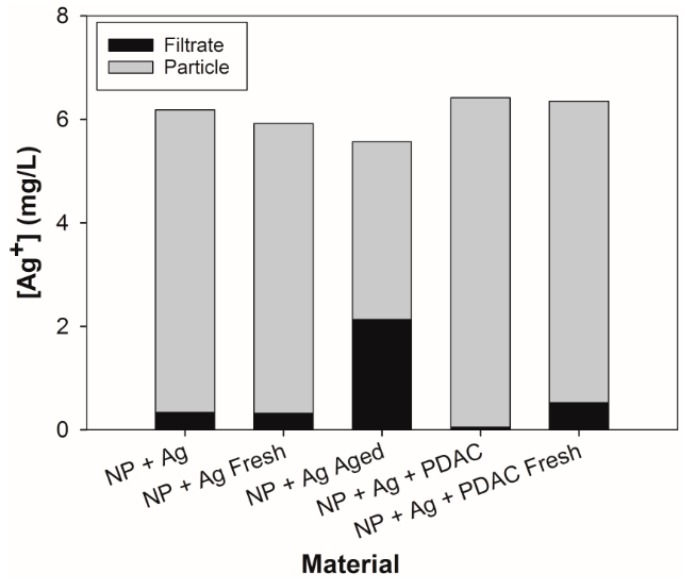
Concentration of silver associated with the filtrate and the particle as determined by ICP analysis. “Fresh” and “Aged” designations relate to the amount of time since the samples were dialyzed. All samples were analyzed via ICP-OES, except for the filtrate in the NP + Ag + PDAC sample, which was analyzed with ICP-MS.

The concentration of silver in solution and the silver associated with the particles was quantified for the five nanoparticle samples that included silver. Figure 1 shows the concentration of silver present in the particle and in the solution which, when combined, matches the nominal concentration provided for each material. In all cases, the silver associated with the particle was greater than the silver present in solution (1.62 to 132 times greater. The full formulation (NP + Ag + PDAC) contains approximately 11 times more silver associated with the particle than the dialyzed full formulation (NP + Ag + PDAC Fresh). Additionally, the age of the NP + Ag sample played a role in silver distribution, as the older formulation contained approximately 10 times more silver in the filtrate than the particle.

Previous analyses of similar particles by Richter and colleagues [35] found that the concentration of silver associated with the particle after dialysis was approximately 18%; however, in this study, we found much higher concentrations associated with the particle (61.7–99.2%). This may have been due to variance between batches of the lignin nanoparticle stock solutions, as well as the differences in digestion techniques. Although the dialysis process is effective at releasing the loosely bound silver from the particles, the electrostatic barrier in the samples that contained PDAC may have impacted the rate at which silver was released. The full formulation had the lowest release of dissolved silver, although there was significantly more silver released from the freshly dialyzed full formulation sample, suggesting that PDAC may retard the release of ionic silver by repulsive electrostatic interactions. Additionally, through previous characterization of the lignin particle functional groups by Richter and colleagues [35,36], there is a higher proportion of organically-bound sulfur compared to other lignin types (nine times that of high-purity lignin), which would likely provide strong binding sites for dissolved silver [45].

### 3.3. Comparative Analysis of Formulation Toxicity

No significant differences were found between replicate exposure plates; therefore, replicates were pooled to increase the sample size to 24 fish per concentration, per material. To encompass all possible comparisons, but for clarity in interpreting the data, two groupings were made, which parallel our hypotheses. These two groupings are formulation comparisons and dialyzed sample comparisons. Concentration-response curves for the two groupings are illustrated in the SI (Figure S3). Additionally, a modeled concentration-response curve was generated for the reference material silver nitrate, which is included in the SI (Figure S4).

#### 3.3.1. Formulation Components

**Figure 2:**
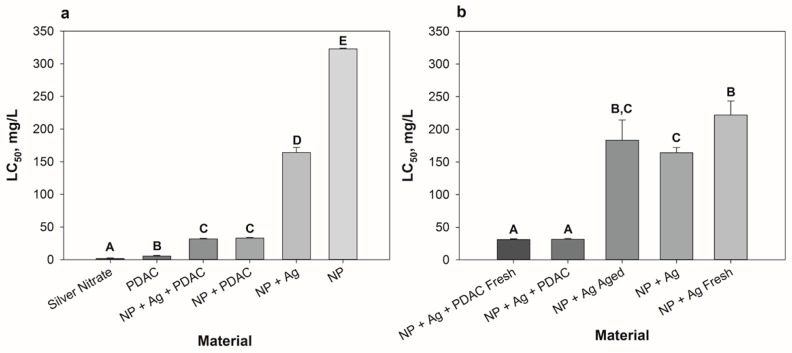
LC_50′_s for nanoparticles and components (**a**) and dialyzed samples (**b**) with standard error of two experimental replicates with 12 embryos exposed at each concentration in each replicate test (24 embryos per concentration total). Significant differences between LC_50_ values are indicated with a change in letter above the bar.

As represented in Figure 2a, the lignin core nanoparticle (NP) itself was the least toxic component (LC_50_ = 323 mg/L), and when the NP was combined with the other aspects of the formulation, LC_50_ values decreased significantly in all cases (NP + Ag LC_50_ = 164 mg/L, NP + PDAC LC_50_ = 33 mg/L, NP + Ag + PDAC LC_50_ = 32 mg/L). The presence of silver in the full formulation (NP + Ag + PDAC) did not change the overall toxicity relative to NP + PDAC. Additionally, when PDAC was present in the formulation (NP + PDAC or NP + Ag + PDAC), a significant increase in mortality events occurred. PDAC and Ag^+^ alone were the two most toxic constituents, with LC_50′_s of 5.39 mg/L and 1.53 mg/L, respectively.

The estimated LC_50_ for Ag^+^ is greater than many published literature values for zebrafish [46,47,48,49,50], however, exposure time and conditions differ in these studies, which may explain the observed differences in toxicity. Our zebrafish embryos were exposed at 8 hpf with the chorion intact, whereas some of the referenced studies did not expose the fish until after hatching, or even as adults. The chorionic membrane can modulate silver toxicity by sequestering ions to prevent them from entering the perivitelline fluid [51], and removing the chorion has been shown to increase toxic responses [40,51]. It is likely that the presence of the chorion may have played a large role in modulating silver toxicity, but the exposure media may have also played a role as well.

The hardness of our prepared fishwater may have altered the toxicity of silver nitrate, as well as nanoparticle-containing formulations to the embryonic zebrafish. Based on dissolved magnesium and calcium concentrations, our fishwater is categorized as soft water (<60 mg/L CaCO_3_), whereas many of the above studies utilize moderately hard to hard water when exposing their zebrafish (up to 148 mg/L CaCO_3_). It has been reported that LC_50′_s tend to be higher in the presence of dissolved organic matter, which has the greatest effect on silver toxicity, followed by Cl^−^, Na^+^, and Ca^2+^ [52]. This is based on the coalescence effect, which leads to complexation and/or formation of nanoparticle agglomerates and/or aggregates, which can decrease apparent toxicity by minimizing particle uptake by the organism [53]. As our fishwater was categorized as soft water (low concentrations of Na^+^ and Ca^2+^), we then determined the concentration of chloride ions present, as silver ion bioavailability can be impacted due to complexation and subsequent precipitation of silver chloride [24,47].

In the Instant Ocean salt formulation used to make the fishwater, the majority of the cations are paired with chloride, and we determined the chloride concentration in our fishwater to be 142 mg/L, which is approximately 55% of the dissolved ion content. It is possible that when the fishwater was used to dilute the silver-containing treatments, the silver complexed with the chloride and precipitated out of solution, which may have led to a greater LC_50_ value. To determine whether precipitation was a significant factor, we utilized Visual MINTEQ (v.3.1) for each silver-containing formulation. We used the filtrate concentrations from the ICP data for each of the formulations that contained silver, and determined that nearly all of the dissolved silver (98.2–99.8%) would complex with the chloride present in the fishwater to form a precipitate of silver chloride, except the NP + Ag + PDAC sample, as the concentration of Ag^+^ in solution was very low. However, as the exposures occurred over a five day period, and the movement of silver from the particle to the surrounding solution is a dynamic process, dissolved silver could have still been bioavailable to the zebrafish.

Considering our nanoparticle and silver combination formulations, comparisons to published LC_50_ values for conventional silver core AgNPs differ significantly. Reported AgNP LC_50′_s for fish generally range between 0.05 and 20 mg/L [2,54]. Variations in reported LC_50_ values may relate to differences in the type of exposure, exposure time, age of the fish, the presence of the chorionic membrane, the use of bare or coated nanoparticles, and/or differences in exposure media. A study completed by Bar-Ilan and colleagues [55] matched our exposure conditions most consistently, and had reported LC_50′_s within the range above, although the LC_50′_s differed from our findings. They exposed embryonic zebrafish to different sizes of colloidal AgNPs (3–100 nm), and found a range of LC_50′_s, from 10.1 to 14.7 mg/L. Although the sizes of the particles, length and timing of the exposure, and retention of the chorionic membrane is consistent with our experiment, our LC_50′_s were an order of magnitude less toxic based on total particle mass (for example, the measured LC_50_ for the NP + Ag formulation was 164 mg/L). Thus, the silver doped lignin nanoparticles have a lower mass-based toxicity than solid core AgNPs, while maintaining a similar antimicrobial efficacy [35]. The difference may be due to the use of the lignin particle, which was shown via ICP-OES/MS analysis to retain bound silver, probably due to the higher binding affinity sites (Figure 1). It should be noted that there was a 100:1 ratio of lignin to Ag^+^ in the nanoparticles, so the amount of silver in each particle was only a hundredth of the mass of the total particle, making our LC_50_ values, based on silver content alone, similar to those measured for solid core AgNPs reported in other studies. Therefore, potential exposure to silver ions would be reduced in the presence of the lignin particles, leading to an apparent increase in the LC_50_. This suggests that by replacing the silver core typical of conventional AgNPs, the concentration of silver released to the environment may be reduced, which was one of the goals of formulating the nanoparticle with a lignin core.

PDAC alone was the second most toxic component tested, and formulations that contained PDAC were significantly more toxic than formulations that did not contain PDAC (Figure 2a). Although PDAC is a high charge density cationic polymer commonly used as a flocculant/coagulant in wastewater treatment, it is also cited as a cytotoxin that interacts with cell membranes to elicit cell damage, and eventually necrosis [56,57]. Our results correspond with this literature finding, as embryos that were exposed to PDAC alone progressively blackened and disintegrated, starting at 5.75 mg/L. Other formulations that contained PDAC did not elicit this response, perhaps because the PDAC is electrostatically associated with the lignin particle, or was “complexed”. Free, or “non-complexed” PDAC, can interact with blood components, such as erythrocytes and plasma proteins, cell membranes, and extracellular matrix proteins, leading to undesired side effects not seen with complexed polycations [58]. Our experimental observations support this, as we see that the uncomplexed PDAC sample is indeed more toxic than any formulation that contains a nanoparticle–PDAC complex (Figure 2a). Additionally, research suggests polycationic polymers like PDAC can disrupt the lipid bilayer, with larger polymers leading to the formation of holes in the lipid membrane that increased membrane permeability [59,60]. Given that information, it is possible that PDAC made the fish more susceptible to both dissolved and particle-bound silver as a result of changes in membrane permeability. The positively charged PDAC-coated particles (SI, Figure S2) may have also been attracted to the negatively-charged membranes of the zebrafish, which could have increased the exposure to silver associated with the particle.

#### 3.3.2. Dialyzed Formulations

The purpose of dialyzing the samples was to simulate post-consumer use of the nanoparticle by purging the surrounding solution of excess silver ions. In the LC_50_ comparisons of dialyzed materials, two results can be observed (Figure 2b). First, there was no difference in LC_50_ between the dialyzed complete formulation (NP + Ag + PDAC Fresh) and its non-dialyzed counterpart (NP + Ag + PDAC). This may be due to similar nominal concentrations of silver, as calculated by adding the silver associated with the particle and silver present in the filtrate (Figure 1). Second, the NP + Ag samples showed a significant decrease in toxicity post-dialysis, with LC_50_ values increasing immediately after dialysis (NP + Ag Fresh, LC_50_ = 222 mg/L), however, the dialyzed aged sample had a similar toxicity to the non-dialyzed sample (NP + Ag LC_50_ = 164 mg/L and NP + Ag Aged LC_50_ = 184 mg/L). Perhaps over time, the higher affinity binding sites on the lignin release more silver into solution compared to the freshly dialyzed sample, leading to the slight decrease in the aged LC_50_ (Figure 1).

### 3.4. Analysis of Sublethal Endpoints

**Figure 3:**
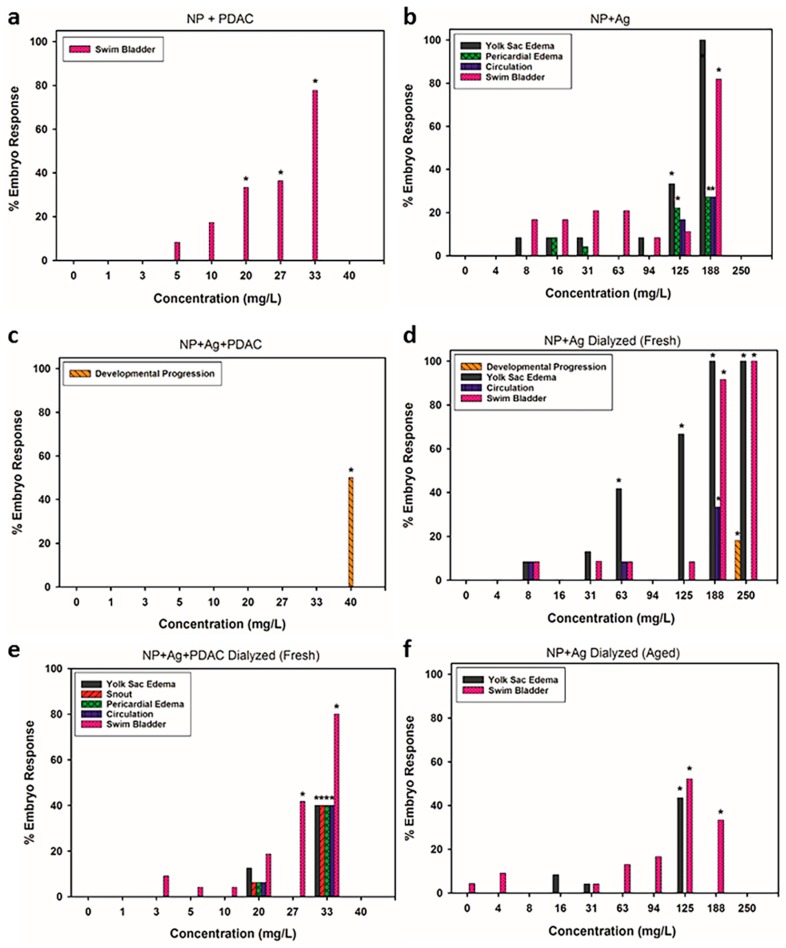
Types of developmental and morphological abnormalities observed in 120 hpf zebrafish embryos exposed to various formulations of the nanoparticles including (**a**) NP + PDAC; (**b**) NP + Ag; (**c**) NP + Ag + PDAC; (**d**) NP + Ag Dialyzed (Fresh); (**e**) NP + Ag + PDAC Dialyzed (Fresh) and (**f**) NP + Ag Dialyzed (Aged). Asterisk represents significant increase relative to unexposed (control) fish embryos at *p* ≤ 0.05.

There were several endpoints that were significant (*p* ≤ 0.05), which included morphological abnormalities, including uninflated swim bladder and snout malformations, developmental endpoints, such as delay in hatching and delayed developmental progression, and physiological anomalies, such as impaired circulation, yolk sac edema, and pericardial edema (Figure 3a–f). Exposure to the silver nitrate at concentrations similar to the amount of silver in the NPs did not cause any significant malformations in the embryos. Overall, there was a significant increase in the types of sublethal responses observed in the dialyzed samples compared to the non-dialyzed samples. The dialyzed samples, particularly the aged sample, had proportionally less silver associated with the particle than the non-dialyzed samples (Figure 1), which could explain the increase in sublethal impacts, as well as the lack of sublethal endpoints for NP + Ag + PDAC (Figure 3).

Swim bladder malformations occurred when silver or PDAC were included in the particle formulation; however, this did not occur when silver and PDAC were both present in the particle formulation (NP + Ag + PDAC), except when freshly dialyzed (Figure 3e). Exposure to silver ions during embryonic zebrafish development has been described as impacting cholinergic signaling, which is important in swim bladder formation [61]. Swim bladder malformations were not significant in the NP + Ag + PDAC sample, likely due to high ratio of silver associated with the particle as compared to the filtrate (Figure 1).

Malformations of the snout were only significant in the freshly dialyzed full formulation (Figure 3e). Although others have reported silver nanoparticles causing snout malformations [54], we do not see this malformation in silver nitrate (Figure S5) or any other formulation containing silver, suggesting some other mechanisms of malformation may be involved. Perhaps the presence of all three formulation components may have contributed to the prevalence of snout malformations, in addition to the increased concentration of silver in the filtrate, as it exceeded the other treatments by a factor of six (Figure 1), except for the aged dialyzed particle (NP + Ag Aged).

Yolk sac edema was present in all fish exposed to formulations containing silver, and impaired circulation was significant in both freshly dialyzed formulations (Figure 3d,e). Significant pericardial edema responses were only noted in the NP + Ag and NP + Ag + PDAC Fresh formulations. Similarly, several other studies have reported that as a result of silver nanoparticle exposure, pericardial edema, yolk sac edema, and impaired circulation are prevalent in the early developmental stages of zebrafish [62,63,64,65]. As the development of the circulatory system and the formation of the heart typically occur between 21 and 24 hpf in zebrafish embryos [42,64], it is likely that the release of dissolved silver from the nanoparticles resulted in these endpoints being prevalent, however, particle-specific responses cannot be ruled out. The chorion has been reported to modulate metal toxicity [51], but there has been recent evidence that nanoparticles (30–72 nm) can move through the chorionic membrane pores and distribute to numerous parts of the fish, including the brain, heart, yolk, and blood [66]. Should the distribution of silver include the yolk and heart, edemas would be expected to occur due to disturbances in osmoregulation [55,62,63,67]. Once these developmental pathways are disturbed during early development, normal embryogenesis can be impacted, resulting in numerous defects [68]. In addition, silver nanoparticles may agglomerate in exposure media, which can alter oxygen exchange through chorionic pores, affecting oxygen tension and osmotic balance, which could then result in edemas similar to those observed in our study [66].

Pericardial edema coupled with impaired circulation following AgNP exposure has been shown to be concentration dependent [63], with an increase in prevalence from 10–100 mg/L. This was the trend we observed in our study, although our study saw increases up to 125 mg/L with a maximum of 17% responding. The most reasonable explanation for the slight difference in observations may be due to differences between solid silver nanoparticles with surface stabilizers, and the lignin-coated particles with specific silver binding sites on the particle core. Polyvinyl alcohol was used as the surface coating in Asharani and colleagues [63], which may have altered the dynamics of silver ion release relative to our samples. Our samples contained PDAC as a surface stabilizer, which provides an electrostatic barrier that could impede silver ion release, which we did see in our samples (Figure 1); however, when freshly dialyzed, PDAC had limited impact on silver ion release, suggesting solution equilibrium may be controlled by PDAC. Additionally, although there was silver present in the filtrate of the freshly dialyzed samples, the lignin core bound the majority of the silver, probably due to the higher affinity silver ion binding sites being utilized.

Significant delay in hatching and delayed developmental progression were the two developmental abnormalities observed in our study following exposure to silver salts (Figure S5) and high concentrations of the bare lignin nanoparticles (Figure S6), respectively. As delayed developmental progression was found following exposure to relatively high concentrations of the bare lignin nanoparticle and no other samples, the bare particle may interact with necessary ions in the solution, making them limited for supporting embryo development at concentrations of 350 mg/L and higher (Figure S6). This is further supported by the finding that this process does not occur when the particle is functionalized with silver, PDAC, or both, as the binding sites on the lignin particle are already occupied. Silver nitrate was the only material tested that led to a delay in hatching. A delay in hatching is primarily caused by deactivation of the ZHE1 enzyme, which prevents chorionic degradation [69]. Lin and colleagues have shown that dissolved metals can interfere with ZHE1, and although silver was included in their assay, it did not lead to a significant decrease in ZHE1 activity [69]. Asharani and colleagues exposed zebrafish to Ag^+^ at 2.14 mg/L, and observed a delay in hatching at 4% compared to controls, but this was not significant [62]. Approximately 50% of our surviving fish exhibited a delay in hatching compared to controls following exposure to silver salts, however, this was not seen in lignin nanoparticle formulations containing silver.

## 4. Conclusions

The results of this study provide several insights into a nanoparticle engineered to be an environmentally friendly alternative to solid silver core nanoparticles. Our data shows that the use of lignin as the nanoparticle core could be a viable alternative, as it did not pose a significant toxicological hazard to our test organism. Since our reported toxicity was similar to other findings when compared on the basis of silver content, the toxicity of silver-enabled nanoparticles may be predictable, based on the silver concentration of the particle. Ionic silver and PDAC alone were the most toxic components of the formulation, which may be attributed to their higher diffusivity and propensity to interact with cell membranes relative to silver and/or PDAC associated with the particle. The inclusion of PDAC not only adds antimicrobial activity to the particle, but also seems to delay the release of silver ions, so in situations where time release of antimicrobial agents is desired, stabilizing the particles with PDAC may be warranted. This data also encourages further development of similar nanomaterials to minimize their impact on the environment, as well as testing the current particle under environmentally relevant conditions to evaluate toxicity. One way of reducing the environmental impact of these engineered nanomaterials is to design them in a way to minimize the release of soluble components, or to replace these components with less toxic ingredients. We are presently investigating the use of an alternative nanoparticle coating which is biologically derived, that may have the potential to be less toxic in comparison to PDAC.

## 5. Associated Content

### Supplemental Information Is Available for This Publication

Representative images of zebrafish with and without significant developmental impacts; Average zeta potential and hydrodynamic diameter measurements for particle-containing formulations over a five day period; Metadata associated with zeta potential measurements; Concentration-response curves for formulation components and dialyzed materials based on zebrafish mortality at 120 hpf; Modeled concentration-response curve for the reference material silver nitrate based on zebrafish mortality at 120 hpf; Visual MINTEQ output for all silver-containing formulations.

## Supplementary Materials

The following are available online at http://www.mdpi.com/2079-6382/7/2/40/s1, Figure S1: Representative images of zebrafish with and without significant developmental impacts, Figure S2: Average zeta potential and hydrodynamic diameter (HDD) of particle formulations over a five day period in fishwater. Figure S1a,b are average zeta potential measurements for the formulation components (a) and the dialyzed formulations (b), and Figure S1c,d are the average HDD measurements for the formulation components (c) and the dialyzed formulations (d), Figure S3: Concentration-response comparisons for formulation components (a) and dialyzed materials (b) based on zebrafish mortality at 120 hpf (a) Significant differences (*p* ≤ 0.05) existed between materials in the formulation component treatments. The lignin particle exhibited the lowest toxicity, followed by silver, and PDAC. (b) No significant differences (*p* > 0.05) existed in the dialyzed sample treatments. Comparisons included the two full formulations (NP + Ag + PDAC) and the three NP + Ag formulations, Figure S4: Concentration–response curve for silver nitrate based on zebrafish mortality at 120 hpf, Figure S5: Frequency of delayed hatching in zebrafish embryos exposed to Ag^+^ as silver nitrate in fishwater. Asterisk represents significant increase (*p* ≤ 0.05) relative to unexposed (control) fish embryos, Figure S6: Frequency of delayed developmental progression in zebrafish embryos exposed to lignin nanoparticles in fishwater. Asterisk represents significant increase (*p* ≤ 0.05) relative to unexposed (control), Table S1: Metadata associated with zeta potential measurements.

## References

[1] Vance M.E., Kuiken T., Vejerano E.P., McGinnis S.P., Hochella M.F., Rejeski D., Hull M.S. (2015). Nanotechnology in the real world: Redeveloping the nanomaterial consumer products inventory. Beilstein J. Nanotechnol..

[2] Bondarenko O., Juganson K., Ivask A., Kasemets K., Mortimer M., Kahru A. (2013). Toxicity of ag, cuo and zno nanoparticles to selected environmentally relevant test organisms and mammalian cells in vitro: A critical review. Arch. Toxicol..

[3] Bystrzejewska-Piotrowska G., Golimowski J., Urban P.L. (2009). Nanoparticles: Their potential toxicity, waste and environmental management. Waste Manag..

[4] Marambio-Jones C., Hoek E.M.V. (2010). A review of the antibacterial effects of silver nanomaterials and potential implications for human health and the environment. J. Nanopart. Res..

[5] Massarsky A., Trudeau V.L., Moon T.W. (2014). Predicting the environmental impact of nanosilver. Environ. Toxicol. Pharmacol..

[6] Piccinno F., Gottschalk F., Seeger S., Nowack B. (2012). Industrial production quantities and uses of ten engineered nanomaterials in europe and the world. J. Nanopart. Res..

[7] Benn T., Cavanagh B., Hristovski K., Posner J.D., Westerhoff P. (2010). The release of nanosilver from consumer products used in the home. J. Environ. Qual..

[8] Benn T.M., Westerhoff P. (2008). Nanoparticle silver released into water from commercially available sock fabrics. Environ. Sci. Technol..

[9] Gottschalk F., Sun T., Nowack B. (2013). Environmental concentrations of engineered nanomaterials: Review of modeling and analytical studies. Environ. Pollut..

[10] Kaegi R., Sinnet B., Zuleeg S., Hagendorfer H., Mueller E., Vonbank R., Boller M., Burkhardt M. (2010). Release of silver nanoparticles from outdoor facades. Environ. Pollut..

[11] Mackevica A., Olsson M.E., Hansen S.F. (2016). The release of silver nanoparticles from commercial toothbrushes. J. Hazard. Mater..

[12] Dobias J., Bernier-Latmani R. (2013). Silver release from silver nanoparticles in natural waters. Environ. Sci. Technol..

[13] Handy R.D., Owen R., Valsami-Jones E. (2008). The ecotoxicology of nanoparticles and nanomaterials: Current status, knowledge gaps, challenges, and future needs. Ecotoxicology.

[14] Maurer-Jones M.A., Gunsolus I.L., Murphy C.J., Haynes C.L. (2013). Toxicity of engineered nanoparticles in the environment. Anal. Chem..

[15] Selck H., Handy R.D., Fernandes T.F., Klaine S.J., Petersen E.J. (2016). Nanomaterials in the aquatic environment: A european union-united states perspective on the status of ecotoxicity testing, research priorities, and challenges ahead: Nanomaterials in the aquatic environment. Environ. Toxicol. Chem..

[16] Furtado L.M., Bundschuh M., Metcalfe C.D. (2016). Monitoring the fate and transformation of silver nanoparticles in natural waters. Bull. Environ. Contam. Toxicol..

[17] Furtado L.M., Norman B.C., Xenopoulos M.A., Frost P.C., Metcalfe C.D., Hintelmann H. (2015). Environmental fate of silver nanoparticles in boreal lake ecosystems. Environ. Sci. Technol..

[18] Kim K.-T., Truong L., Wehmas L., Tanguay R.L. (2013). Silver nanoparticle toxicity in the embryonic zebrafish is governed by particle dispersion and ionic environment. Nanotechnology.

[19] Peijnenburg W.J.G.M., Baalousha M., Chen J., Chaudry Q., Von der kammer F., Kuhlbusch T.A.J., Lead J., Nickel C., Quik J.T.K., Renker M. (2015). A review of the properties and processes determining the fate of engineered nanomaterials in the aquatic environment. Crit. Rev. Environ. Sci. Technol..

[20] Lacave J.M., Retuerto A., Vicario-Parés U., Gilliland D., Oron M., Cajaraville M.P., Orbea A. (2016). Effects of metal-bearing nanoparticles (Ag, Au, CdS, ZnO, SiO_2_) on developing zebrafish embryos. Nanotechnology.

[21] Nel A., Xia T., Mädler L., Li N. (2006). Toxic potential of materials at the nanolevel. Science.

[22] Sharma V.K., Siskova K.M., Zboril R., Gardea-Torresdey J.L. (2014). Organic-coated silver nanoparticles in biological and environmental conditions: Fate, stability and toxicity. Adv. Colloid Interface Sci..

[23] Shin S., Song I., Um S. (2015). Role of physicochemical properties in nanoparticle toxicity. Nanomaterials.

[24] Groh K.J., Dalkvist T., Piccapietra F., Behra R., Suter M.J.F., Schirmer K. (2015). Critical influence of chloride ions on silver ion-mediated acute toxicity of silver nanoparticles to zebrafish embryos. Nanotoxicology.

[25] Ivask A., ElBadawy A., Kaweeteerawat C., Boren D., Fischer H., Ji Z., Chang C.H., Liu R., Tolaymat T., Telesca D. (2014). Toxicity mechanisms in escherichia coli vary for silver nanoparticles and differ from ionic silver. ACS Nano.

[26] Clement J., Jarrett P. (1994). Antibacterial silver. Met. Based Drugs.

[27] Gordon O., Vig Slenters T., Brunetto P.S., Villaruz A.E., Sturdevant D.E., Otto M., Landmann R., Fromm K.M. (2010). Silver coordination polymers for prevention of implant infection: Thiol interaction, impact on respiratory chain enzymes, and hydroxyl radical induction. Antimicrob. Agents Chemother..

[28] Morones J.R., Elechiguerra J.L., Camacho A., Holt K., Kouri J.B., Ramírez J.T., Yacaman M.J. (2005). The bactericidal effect of silver nanoparticles. Nanotechnology.

[29] Feng Q.L., Wu J., Chen G.Q., Cui F.Z., Kim T.N., Kim J.O. (2000). A mechanistic study of the antibacterial effect of silver ions on escherichia coli and staphylococcus aureus. J. Biomed. Mater. Res..

[30] Fong J., Wood F. (2006). Nanocrystalline silver dressings in wound management: A review. Int. J. Nanomed..

[31] Hwang E.T., Lee J.H., Chae Y.J., Kim Y.S., Kim B.C., Sang B.-I., Gu M.B. (2008). Analysis of the toxic mode of action of silver nanoparticles using stress-specific bioluminescent bacteria. Small.

[32] Sharma V.K., Yngard R.A., Lin Y. (2009). Silver nanoparticles: Green synthesis and their antimicrobial activities. Adv. Colloid Interface Sci..

[33] Sabella S., Carney R.P., Brunetti V., Malvindi M.A., Al-Juffali N., Vecchio G., Janes S.M., Bakr O.M., Cingolani R., Stellacci F. (2014). A general mechanism for intracellular toxicity of metal-containing nanoparticles. Nanoscale.

[34] Anastas P., Eghbali N. (2010). Green chemistry: Principles and practice. Chem. Soc. Rev..

[35] Richter A.P., Brown J.S., Bharti B., Wang A., Gangwal S., Houck K., Cohen Hubal E.A., Paunov V.N., Stoyanov S.D., Velev O.D. (2015). An environmentally benign antimicrobial nanoparticle based on a silver-infused lignin core. Nat. Nanotechnol..

[36] Richter A.P., Bharti B., Armstrong H.B., Brown J.S., Plemmons D., Paunov V.N., Stoyanov S.D., Velev O.D. (2016). Synthesis and characterization of biodegradable lignin nanoparticles with tunable surface properties. Langmuir.

[37] Frangville C., Rutkevičius M., Richter A.P., Velev O.D., Stoyanov S.D., Paunov V.N. (2012). Fabrication of environmentally biodegradable lignin nanoparticles. ChemPhysChem.

[38] Hill A.J. (2005). Zebrafish as a model vertebrate for investigating chemical toxicity. Toxicol. Sci..

[39] Truong L., Harper S., Tanguay R. (2011). Evaluation of embryotoxicity using the zebrafish model. Drug Safety Evaluation: Methods and Protocols.

[40] Kim K.-T., Tanguay R.L. (2014). The role of chorion on toxicity of silver nanoparticles in the embryonic zebrafish assay. Environ. Health Toxicol..

[41] Duval A., Lawoko M. (2014). A review on lignin-based polymeric, micro- and nano-structured materials. React. Funct. Polym..

[42] Kimmel C.B., Ballard W.W., Kimmel S.R., Ullmann B., Schilling T.F. (1995). Stages of embryonic development of the zebrafish. Dev. Dyn..

[43] Wu F., Harper B.J., Harper S.L. (2017). Differential dissolution and toxicity of surface functionalized silver nanoparticles in small-scale microcosms: Impacts of community complexity. Environ. Sci. Nano.

[44] Sprague J., Fogels A. (1977). Watch the y in Bioassay.

[45] Bielmyer G.K., Grosell M., Paquin P.R., Mathews R., Wu K.B., Santore R.C., Brix K.V. (2007). Validation study of the acute biotic ligand model for silver. Environ. Toxicol. Chem..

[46] Alsop D., Wood C.M. (2011). Metal uptake and acute toxicity in zebrafish: Common mechanisms across multiple metals. Aquat. Toxicol..

[47] Bielmyer G., Brix K., Grosell M. (2008). Is Cl^−^ protection against silver toxicity due to chemical speciation?. Aquat. Toxicol..

[48] Bilberg K., Hovgaard M.B., Besenbacher F., Baatrup E. (2012). In vivo toxicity of silver nanoparticles and silver ions in zebrafish (*Danio rerio*). J. Toxicol..

[49] Powers C.M., Slotkin T.A., Seidler F.J., Badireddy A.R., Padilla S. (2011). Silver nanoparticles alter zebrafish development and larval behavior: Distinct roles for particle size, coating and composition. Neurotox. Teratol..

[50] Powers C.M., Yen J., Linney E.A., Seidler F.J., Slotkin T.A. (2010). Silver exposure in developing zebrafish (*danio rerio*): Persistent effects on larval behavior and survival. Neurotoxicol. Teratol..

[51] Rombough P. (1985). The influence of zona radiata on the toxicities of zinc, lead, mercury, copper and silver ions to embryos of steelhead trout salmo gairdneri. Comp. Biochem. Physiol..

[52] McGeer J.C., Playle R.C., Wood C.M., Galvez F. (2000). A physiologically based biotic ligand model for predicting the acute toxicity of waterborne silver to rainbow trout in freshwaters. Environ. Sci. Technol..

[53] Lapresta-Fernández A., Fernández A., Blasco J. (2012). Nanoecotoxicity effects of engineered silver and gold nanoparticles in aquatic organisms. TrAC Trends Anal. Chem..

[54] Reidy B., Haase A., Luch A., Dawson K., Lynch I. (2013). Mechanisms of silver nanoparticle release, transformation and toxicity: A critical review of current knowledge and recommendations for future studies and applications. Materials.

[55] Bar-Ilan O., Albrecht R.M., Fako V.E., Furgeson D.Y. (2009). Toxicity assessments of multisized gold and silver nanoparticles in zebrafish embryos. Small.

[56] Fischer D., Li Y., Ahlemeyer B., Krieglstein J., Kissel T. (2003). In vitro cytotoxicity testing of polycations: Influence of polymer structure on cell viability and hemolysis. Biomaterials.

[57] Wandrey C., Hernandez-Barajas J., Hunkeler D. (1999). Diallyldimethylammonium chloride and its polymers. Adv. Polym. Sci..

[58] Kircheis R., Wightman L., Wagner E. (2001). Design and gene delivery activity of modified polyethylenimines. Adv. Drug Del. Rev..

[59] Hong S., Leroueil P.R., Janus E.K., Peters J.L., Kober M.-M., Islam M.T., Orr B.G., Baker J.R., Banaszak Holl M.M. (2006). Interaction of polycationic polymers with supported lipid bilayers and cells: Nanoscale hole formation and enhanced membrane permeability. Bioconjug. Chem..

[60] Mecke A., Majoros I.J., Patri A.K., Baker J.R., Banaszak Holl M.M., Orr B.G. (2005). Lipid bilayer disruption by polycationic polymers: The roles of size and chemical functional group. Langmuir.

[61] Robertson G.N., McGee C.A.S., Dumbarton T.C., Croll R.P., Smith F.M. (2007). Development of the swimbladder and its innervation in the zebrafish,danio rerio. J. Morphol..

[62] Asharani P.V., Lian Wu Y., Gong Z., Valiyaveettil S. (2008). Toxicity of silver nanoparticles in zebrafish models. Nanotechnology.

[63] Asharani P.V., Lian Wu Y., Gong Z., Valiyaveettil S. (2011). Comparison of the toxicity of silver, gold and platinum nanoparticles in developing zebrafish embryos. Nanotoxicology.

[64] Lee K.J., Browning L.M., Nallathamby P.D., Osgood C.J., Xu X.-H.N. (2013). Silver nanoparticles induce developmental stage-specific embryonic phenotypes in zebrafish. Nanoscale.

[65] Osborne O.J., Johnston B.D., Moger J., Balousha M., Lead J.R., Kudoh T., Tyler C.R. (2013). Effects of particle size and coating on nanoscale ag and tio2 exposure in zebrafish (*danio rerio*) embryos. Nanotoxicology.

[66] Liu W., Long Y., Yin N., Zhao X., Sun C., Zhou Q., Jiang G. (2016). Toxicity of engineered nanoparticles to fish. Engineered Nanoparticles and the Environment: Biophysicochemical Processes and Toxicity.

[67] Lee K.J., Nallathamby P.D., Browning L.M., Osgood C.J., Xu X.-H.N. (2007). In vivo imaging of transport and biocompatibility of single silver nanoparticles in early development of zebrafish embryos. ACS Nano.

[68] Kiener T.K., Selptsova-Friedrich I., Hunziker W. (2008). TJP3/ZO-3 is critical for epidermal barrier function in zebrafish embryos. Dev. Biol..

[69] Lin S., Zhao Y., Ji Z., Ear J., Chang C.H., Zhang H., Low-Kam C., Yamada K., Meng H., Wang X. (2013). Zebrafish high-throughput screening to study the impact of dissolvable metal oxide nanoparticles on the hatching enzyme, ZHE1. Small.

