# Supplementary material for: Toxicological Assessment of a Lignin Core Nanoparticle Doped with Silver as an Alternative to Conventional Silver Core Nanoparticles
Source: Antibiotics (Basel). 2018 May 4;7(2):40. doi: 10.3390/antibiotics7020040 (PMC6023088; doi:10.3390/antibiotics7020040)
Supplement: Supplementary file 1 [file antibiotics-07-00040-s001.pdf]

antibiotics
MDPI

Toxicological Assessment of a Lignin Core Nanoparticle Doped with Silver as an Alternative to Conventional Silver Core Nanoparticles

Cassandra E. Nix, Bryan J. Harper, Cathryn G. Conner, Alexander P. Richter, Orlin D. Velev & Stacey L. Harper

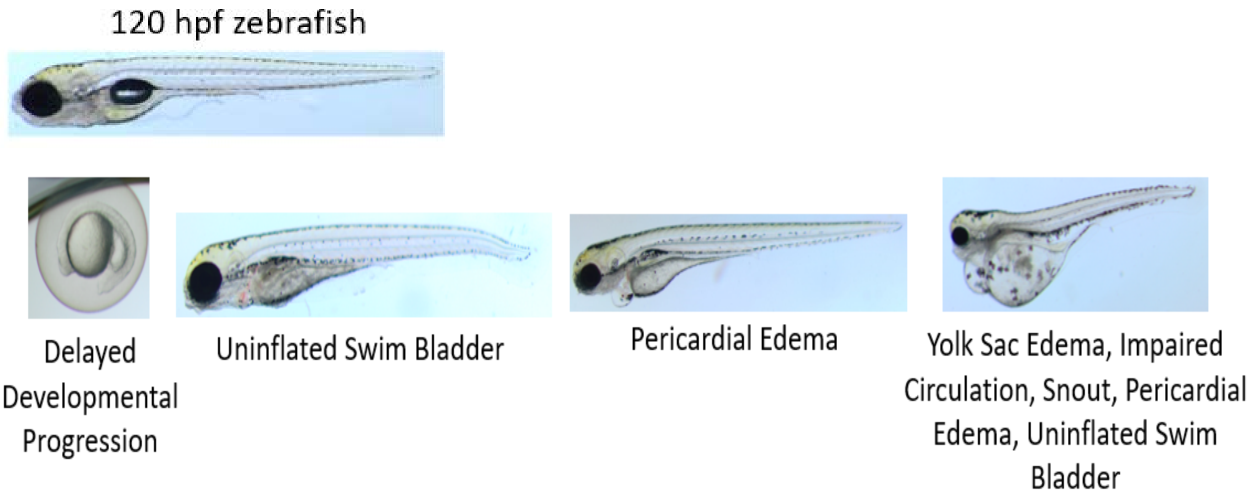

Figure S1. Representative images of zebrafish with and without significant developmental impacts.

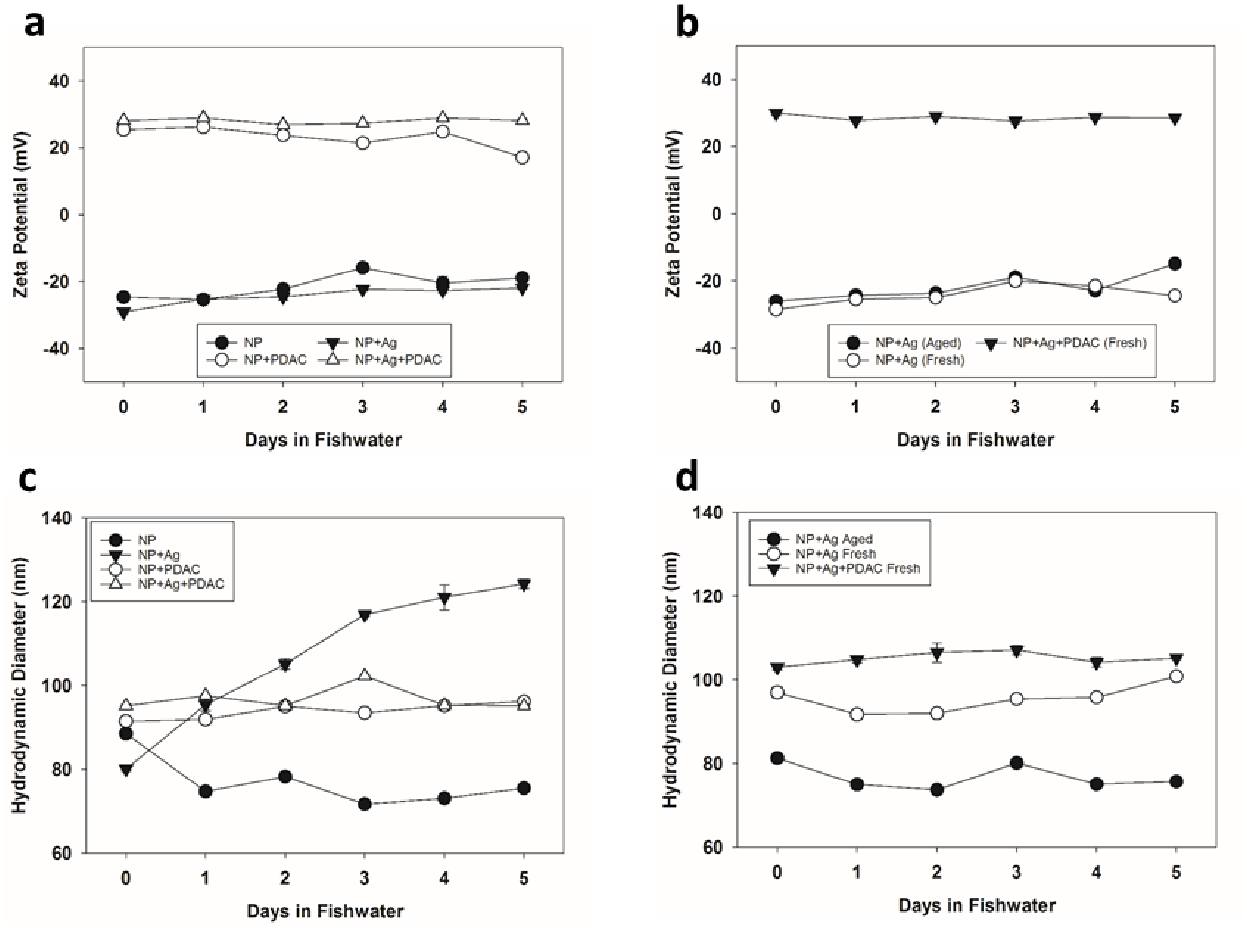

Figure S2. Average zeta potential and hydrodynamic diameter (HDD) of particle formulations over a five day period in fishwater. Figures S1 a–b are average zeta potential measurements for the formulation components (a) and the dialyzed formulations (b), and figures S1 c–d are the average HDD measurements for the formulation components (c) and the dialyzed formulations (d).

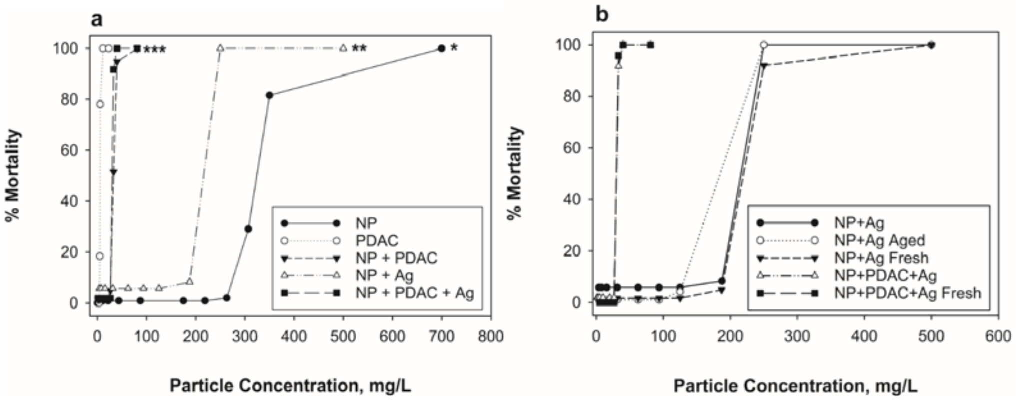

Figure S3. Concentration−response comparisons for formulation components (a) and dialyzed materials (b) based on zebrafish mortality at 120 hpf. (a) Significant differences ($p \leq 0.05$) existed between materials in the formulation component treatments. The lignin particle exhibited the lowest toxicity, followed by silver, and PDAC. (b) No significant differences ($p > 0.05$) existed in the dialyzed sample treatments. Comparisons included the two full formulations (NP+Ag+PDAC) and the three NP+Ag formulations.

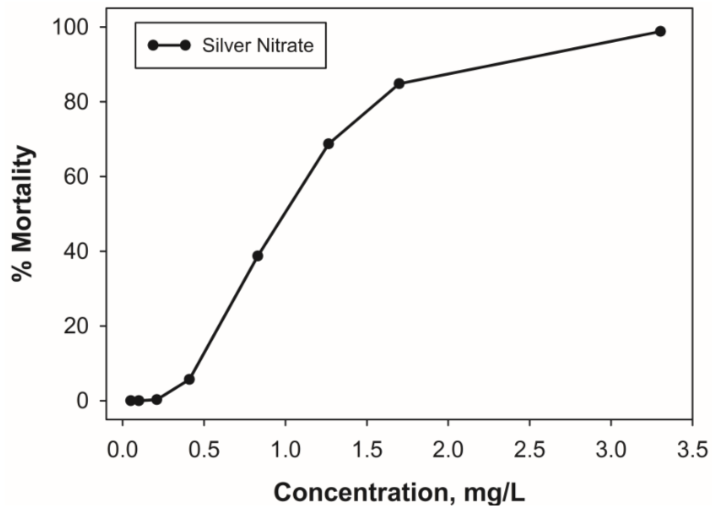

Figure S4. Concentration−response curve for silver nitrate based on zebrafish mortality at 120 hpf.

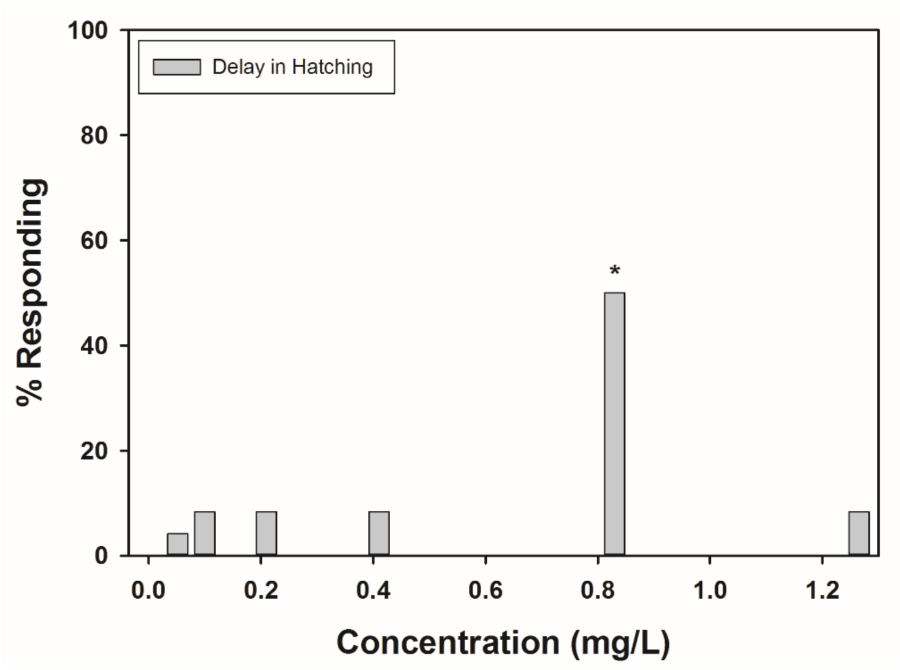

Figure S5. Frequency of delayed hatching in zebrafish embryos exposed to Ag^+ as silver nitrate in fishwater. Asterisk represents significant increase ($p \leq 0.05$) relative to unexposed (control) fish embryos.

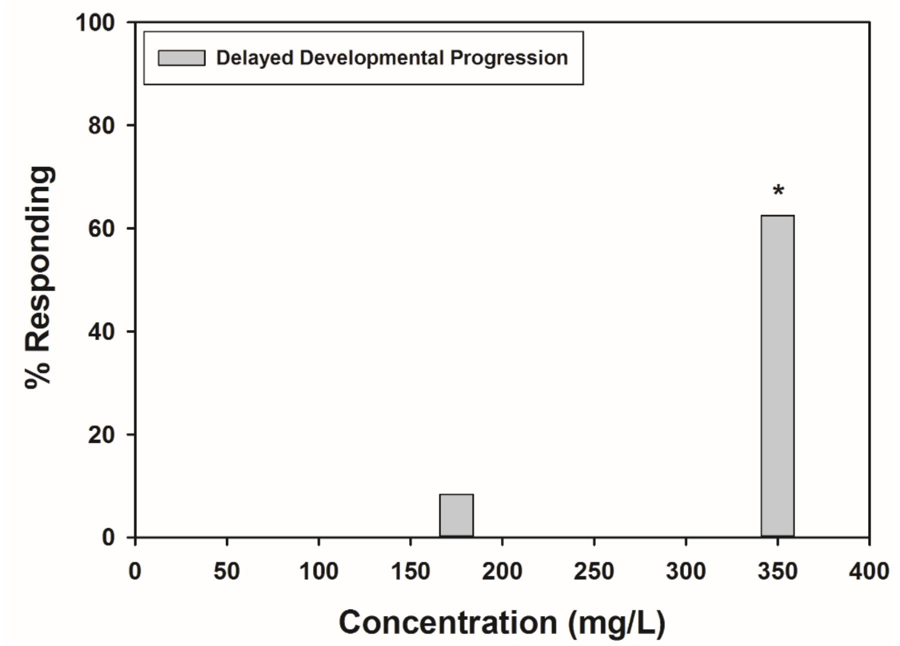

Figure S6. Frequency of delayed developmental progression in zebrafish embryos exposed to lignin nanoparticles in fishwater. Asterisk represents significant increase ($p \leq 0.05$) relative to unexposed (control).

Table S1. Metadata associated with zeta potential measurements.

Model Used to Compute the Zeta Potential	Smoluchowski Equation
pH	7.2

Ionic strength	260 mg/L
Ionic composition	Made per reference 38
Temperature	26.8 °C
Viscosity	0.8508 mPa
Macromolecules/Natural Organic Matter	None
Duration of Measurement	1 minute
Applied voltage	148 V
Number of instrument measurements made and averaged to determine ZP	12
Total number of replicate measurements	3
